# Raloxifene inhibits pancreatic adenocarcinoma growth by interfering with ERβ and IL-6/gp130/STAT3 signaling

**DOI:** 10.1007/s13402-020-00559-9

**Published:** 2020-09-17

**Authors:** Ioannis Pozios, Nina N. Seel, Nina A. Hering, Lisa Hartmann, Verena Liu, Peter Camaj, Mario H. Müller, Lucas D. Lee, Christiane J. Bruns, Martin E. Kreis, Hendrik Seeliger

**Affiliations:** 1 Department of General, Visceral and Vascular Surgery, Campus Benjamin Franklin, Charité Universitätsmedizin Berlin, Hindenburgdamm 30, 12203 Berlin, Germany; 2 Department of General, Visceral and Transplantation Surgery, Hospital of the University of Munich, Munich, Germany; 3 Department of Minimal Invasive and Visceral Surgery, Vivantes Klinikum Neukölln, Berlin, Germany; 4 Department of General, Visceral, Cancer and Transplant Surgery, University Hospital of Cologne, Cologne, Germany

**Keywords:** Pancreatic cancer, Estrogen receptor, SERM, Raloxifene, ERβ, IL-6, gp130, STAT3

## Abstract

**Purpose:**

Currently, the exact role of estrogen receptor (ER) signaling in pancreatic cancer is unknown. Recently, we showed that expression of phosphorylated ERβ correlates with a poor prognosis in patients with pancreatic ductal adenocarcinoma (PDAC). Here, we hypothesized that raloxifene, a FDA-approved selective ER modulator (SERM), may suppress PDAC tumor growth by interfering with ERβ signaling. To test this hypothesis, we studied the impact of raloxifene on interleukin-6/glycoprotein-130/signal transducer and activator of transcription-3 (IL-6/gp130/STAT3) signaling.

**Methods:**

Human PDAC cell lines were exposed to raloxifene after which growth inhibition was assessed using a BrdU assay. ER knockdown was performed using siRNAs specific for ERα and ERβ. The effects of raloxifene on IL-6 expression and STAT3 phosphorylation in PDAC cells were assessed by ELISA and Western blotting, respectively. In addition, raloxifene was administered to an orthotopic PDAC tumor xenograft mouse model, after which tumor growth was monitored and immunohistochemistry was performed.

**Results:**

Raloxifene inhibited the in vitro growth of PDAC cells, and this effect was reversed by siRNA-mediated knockdown of ERβ, but not of ERα, indicating ER isotype-specific signaling. We also found that treatment with raloxifene inhibited the release of IL-6 and suppressed the phosphorylation of STAT3^Y705^ in PDAC cells. In vivo, we found that orthotopic PDAC tumor growth, lymph node and liver metastases as well as Ki-67 expression were reduced in mice treated with raloxifene.

**Conclusions:**

Inhibition of ERβ and the IL-6/gp130/STAT3 signaling pathway by raloxifene leads to potent reduction of PDAC growth in vitro and in vivo. Our results suggest that ERβ signaling and IL-6/gp130 interaction may serve as promising drug targets for pancreatic cancer and that raloxifene may serve as an attractive therapeutic option for PDAC patients expressing the ERβ isotype.

## Introduction

Pancreatic ductal adenocarcinoma (PDAC) is a highly lethal disease with a dismal prognosis and a mortality rate closely paralleling its incidence [1]. The total number of deaths from pancreatic cancer is currently increasing and has been predicted to be the second leading cause of cancer death in the USA by 2030 [2]. In spite of refined surgical and non-surgical treatment options that have been developed in the last decade, little success has been achieved in improving the survival of patients suffering from PDAC.

When characterizing molecular targets for potential prognostic and therapeutic use, differences in gender distribution have led to assessment of the role of estrogen receptors (ERs) in the development and progression of PDAC. Epidemiological data have suggested a protective effect of estrogen for pancreatic cancer and, consequently, the incidence of PDAC has been found to be higher in men than in premenopausal women, with the gender difference decreasing after menopause [3, 4]. Since the first report of ER expression in pancreatic cancer [5], several studies have been reported on the role of ER expression and targeting in PDAC, leading to ambivalent results [6–11]. Previously, we found that expression of ER beta (ERβ) and its phosphorylated form may serve as an independent prognostic marker in PDAC patients and is correlated with a poor prognosis [12, 13]. These findings led to the hypothesis that targeting ERβ may result in reduced PDAC progression and metastasis. The objective of the present study was to identify inhibitory effects of raloxifene, a non-steroidal selective estrogen receptor modulator (SERM) with ERβ-antagonistic properties, on PDAC cells in vitro and in vivo. Furthermore, we aimed to identify inhibitory effects of raloxifene on the interleukin-6/glycoprotein-130/signal transducer and activator of transcription 3 (IL-6/gp130/STAT3) pathway that has been shown to interact with ER signaling and to represent one of the essential signaling cascades in PDAC initiation and progression [14].

## Materials and methods

### Cell lines and reagents

The human PDAC cell lines L3.6pl and AsPC-1 were used for in vitro and in vivo studies. AsPC-1 cells were obtained from the American Type Culture Collection (ATCC; Rockville, MD, USA). L3.6pl is a secondary highly metastatic human pancreatic adenocarcinoma cell line derived from an orthotopic mouse xenograft model [15]. All cell lines were maintained in Dulbecco’s Minimal Essential Medium (Invitrogen GmbH, Karlsruhe, Germany), supplemented with 10% fetal bovine serum and 1% penicillin streptomycin and were incubated in a humidified atmosphere of 5% CO_2_ at 37 °C.

Raloxifene was purchased from Sigma-Aldrich (Schnelldorf, Germany) and was dissolved in 0.1% dimethyl sulfoxide (DMSO, Carl Roth GmbH & Co. KG, Karlsruhe, Germany). IL-6 was purchased from Invitrogen GmbH (Karlsruhe, Germany) and was dissolved in acetic acid. Anti-STAT3, anti-phosphorylated-STAT3 (Y705) and anti-gp130 antibodies were obtained from Cell Signaling Technology (Frankfurt am Main, Germany).

### Cell proliferation assay

L3.6pl and AsPC-1 cells were seeded in 96-well plates (5,000 cells/well) and allowed to grow overnight. Next, cells were incubated with raloxifene in serum-free medium at 37 °C for 48 hours. Cell proliferation was determined using a 5-bromo-2′-deoxyuridine (BrdU) incorporation assay according to the manufacturer’s instructions (Roche Applied Science, Penzberg, Germany). All assays were conducted in quintuples.

### ER silencing in PDAC cells

For in vitro ER knockdown assessment, RNA was isolated from AsPC-1 and L3.6pl cells using a RNeasy™ Mini Kit (Qiagen; Leipzig, Germany). Reverse transcription was carried out using a SuperScript™ III One-Step RT-PCR System with Platinum™ Taq High Fidelity (Thermo Fisher Scientific, Bremen, Germany) according to the manufacturer’s instructions. ERβsi forward and ERβsi reverse primers were developed in such a way that they amplified 586 bp or 531 bp, respectively. ERαsi: for 5’- GTATGATCCTACCAGACCCTTCAGTGA-3’; rev 5’-AGATGCTCCATGCCTTTGTTAC TC-3’. ERβsi: for 5’-GCCGCCCCATGTGCTGAT-3’; rev 5’-ATGGATTGCTGCTGGGAG GAGATG-3’. siRNAs were generated using BLOCK-iT™ RNAi TOPO Transcription Kits and BLOCK-iT™ Dicer RNAi Kits (both Thermo Fisher Scientific) according to the manufacturer’s instructions. Lipofectamine™ 2000 (Thermo Fisher Scientific) was used for the transfection of L3.6pl cells with the designed siRNAs. Control cells were transfected with siRNAs specific for the lacZ reporter gene, which was generated in the same way, using primers and templates supplied with the kit.

### Enzyme-linked immunosorbent assay (ELISA)

Interleukin-6 concentrations in the cell culture supernatants were determined by ELISA (human IL-6 ELISA, BenderMed Systems, Vienna, Austria) according to the manufacturer’s instructions. 200,000 L3.6pl cells per well were seeded into 6-well plates and stimulated with lipopolysaccharide (LPS; Sigma-Aldrich, Taufkirchen, Germany) for 24 hours. After the incubation phase, raloxifene was added for an additional 48 hours. Next, the supernatants were removed and centrifuged at 1700 g for 10 minutes. All ELISA assays were performed in quintuples.

### Western blotting

PDAC cells were treated with different concentrations of raloxifene or solvent in serum-free medium. Phosphorylation was stimulated by IL-6 two hours after the addition of raloxifene. After an incubation period of three, six, or 24 hours, cells were washed with ice-cold phosphate buffer saline (PBS) and lysed in radioimmunoprecipitation assay lysis buffer containing phosphatase and protease inhibitors. Protein concentrations were determined using a Quantipro™ BCA (bicinchoninic acid) protein assay kit (Sigma-Aldrich, Taufkirchen, Germany) according to the manufacturer’s instructions. Next, the proteins were separated by sodium dodecyl sulfate polyacrylamide gel electrophoresis and electro-transferred to polyvinylidene difluoride membranes (Perkin Elmer, Boston, USA). The resulting membranes were blocked (Tris-buffered saline with Tween-80, 5% bovine serum albumin and 0,02% sodium azide) and incubated with specific primary antibodies overnight at 4 °C and, subsequently, probed with horseradish peroxidase-conjugated secondary antibodies. Signals were visualized by luminescence imaging (Peqlab Biotechnologie GmbH, Erlangen, Germany) using an enhanced chemiluminescent substrate system (SuperSignal™ West Pico Plus, Thermo Fisher, Ulm, Germany).

### DARTS assay

DARTS (Drug Affinity Responsive Target Stability) assays, which identify potential protein targets for small molecules via protease protection from pronase, were carried out as described previously [16, 17]. Briefly, L3.6pl cells were lysed using M-PER™ (Thermo Fisher) supplemented with protease and phosphatase inhibitors. Cell lysates containing 2 to 5 µg/µl total proteins were treated with 4 µM raloxifene or the direct gp130-inhibitor SC144 at room temperature for 60 minutes, followed by proteolysis for five minutes at room temperature with pronase (1:10,000 ratio; Roche Applied Science, Penzberg, Germany), as described previously [16, 17]. Proteolysis was stopped by adding SDS loading buffer and heating to 95 °C for five minutes. Finally, the samples were analyzed by Western blotting as described above.

### In vivo orthotopic xenograft experiments

Male, 6–8 week old nude mice (Balb/c nu/nu) were purchased from Charles River (Sulzfeld, Germany) and were kept in air-conditioned rooms at 20 ± 2 °C with a 12-hour light-dark period under specific pathogen-free conditions. Anesthesia, laparotomy and pancreas mobilization were performed as described previously [15, 18]. One million L3.6pl or AsPC-1 cells were suspended in PBS and injected into the pancreatic tail of each animal. The pancreas was subsequently repositioned and the abdomen was closed. The mice were randomized into two groups with 10 mice per group. Two days after orthotopic injection of the tumor cells, treatment was started by daily intraperitoneal injection of raloxifene (30 mg per kg of body weight) or vehicle (Corn Oil, Sigma-Aldrich) at the same volume for a total of 30 days. After treatment, all animals were sacrificed, and the tumors were removed. The tumor volumes were calculated according to the ellipsoid volume formula (tumor volume = length x width x height / 2). The tumors were fixed in formalin, embedded in paraffin, and sectioned for immunohistochemical analysis. All animal experiments were approved by the District Government of Upper Bavaria, Munich, Germany. Animal care and manipulation were in agreement with the European Union Guidelines on Animal Experiments.

### Immunohistochemistry (IHC)

Paraffin-embedded tissues were used for the assessment of Ki67, CD31 (PECAM-1), ERα and ERβ expression. Anti-ERα, anti-ERβ and anti-Ki67 antibodies were purchased from Abcam (Cambridge, UK), anti-CD31 was purchased from Cell Signalling (Cambridge, UK). 

All sections were first deparaffinized in Neo-Clear™ (Merck, Darmstadt, Germany) and rehydrated in a graded series of alcohol. Tissue sections were subsequently blocked by 8% bovine serum albumin using an Avidin/Biotin blocking kit (Vector Laboratories, Burlingame, CA, USA), followed by incubation with the indicated antibody at 4 °C overnight. Next, tissue sections were incubated with a biotin-conjugated secondary antibody (Dako GmbH, Hamburg, Germany) using an Avidin-Biotin complex kit (Vector Laboratories, Burlingame, CA, USA). DAB + chromogen (3,3 diaminobenzidine; Dako GmbH, Hamburg, Germany) was used to generate fluorescent signals, whereas counterstaining was carried out using a hemalum solution (Merck, Darmstadt, Germany). Immunoperoxidase-based detection was performed using HistoMark^®^ RED (Sera Care, Milford, USA). 

Microscopy was performed using a 400x magnification and ten fields of each section were pictured by Axiocam 305 color (Zeiss, Jena, Germany). The area of each pictured field was 0.0952 mm^2^. For quantification of Ki67 staining, the number of Ki67 positive and negative cells were counted in each field and percentages of positive cells were calculated. For the quantification of microvessel density, the number of vessels was counted in ten areas exhibiting most intense CD31 expression. The mean was calculated as the number of microvessels per area (0.0952 mm^2^). All values are given as mean ± SEM.

### Statistical analysis

Statistical analyses and *p*-value determinations were carried out by t-test with a confidence interval of 95% for determination of significant differences between treatment groups. A *p*-value of < 0.05 was considered statistically significant. SPSS software, version 20.0 (IBM Corp., Armonk, NY, USA) was used for the statistical analyses.

## Results

### Raloxifene inhibits pancreatic cancer cell proliferation in a dose-dependent manner in vitro

**Fig. 1:**
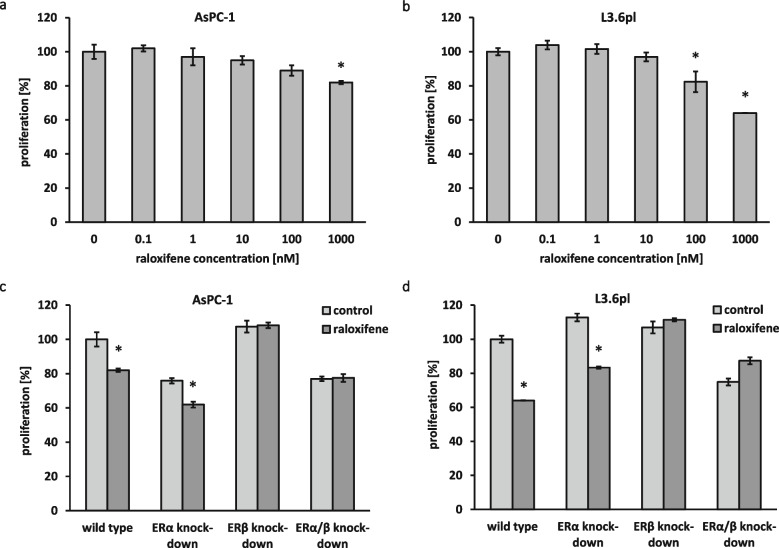
Raloxifene inhibits AsPC-1 and L3.6pl pancreatic cancer cell proliferation in a dose-dependent and ERβ-dependent manner. BrdU proliferation assays under raloxifene treatment in different concentrations in ER-wild type AsPC-1 (**a**) and L3.6pl (**b**) pancreatic cancer cells. Inhibition of AsPC-1 and L3.6pl cell proliferation by raloxifene (1 µM) was ERβ-dependent. Note that in both constellations in which ERβ expression was silenced (ERβ knock-down and combined ERα/ERβ knock-down), no effect of raloxifene was observed (**c** and **d**); * *p* < 0.05

Tumor growth largely depends on the proliferation of cancer cells. Taken into account the previously reported proliferation-inhibiting effects of SERMs on breast cancer cells [19], the effect of SERM raloxifene on the proliferation of AsPC-1 and L3.6pl pancreatic cancer cells was assessed using a BrdU proliferation assay. We found that treatment with raloxifene for 48 hours induced a dose-dependent antiproliferative effect in both cell lines (Fig. 1a and b).

### Inhibition of pancreatic cancer cell proliferation by raloxifene is dependent on ERβ

In order to find out whether the inhibitory effect of raloxifene on PDAC cell proliferation depends on the presence of ERs, RNA interference (RNAi) was applied. After transient transfection with ERα or ERβ specific siRNAs, the expression of ERα and ERβ in AsPC-1 and L3.6pl cells was found to be suppressed. Since raloxifene interacts with both receptors, both single and double inhibition of ERα and ERβ was performed to be able to uncover possible receptor-unspecific effects. In both constellations in which ERβ expression was reduced to a minimum (i.e., silencing ERβ alone or combined ERα and ERβ silencing), no cell proliferation inhibition by raloxifene was observed (Fig. 1c and d).

### Raloxifene suppresses IL-6 secretion in human pancreatic cancer cells

**Fig. 2:**
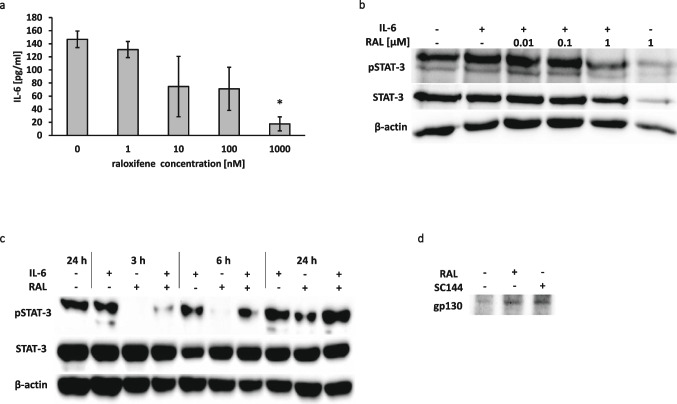
Raloxifene suppresses IL-6 secretion and IL-6-induced STAT3 phosphorylation in L3.6pl pancreatic cancer cells. ELISA showing that IL-6 concentrations are reduced after 24 hours of raloxifene treatment at different concentrations (**a**) (* *p* < 0.05). Representative Western blots showing that raloxifene inhibits IL-6-induced STAT3 phosphorylation in L3.6pl cells in a dose- and time-dependent manner (**b** and **c**, respectively). DARTS assay showing protein-ligand interaction between raloxifene and gp130. SC144 is a known gp130 inhibitor and was used as a positive control. The affinity of raloxifene to gp130 was similar to that of SC144 at a pronase concentration of 1:10,000 (**d**)

Since we found that raloxifene exerts inhibitory effects on the proliferation of human PDAC cells, an explanatory starting-point for its mechanism of action was sought for. Therefore, a specific sandwich ELISA was carried out to verify an anticipated inhibitory effect on the distribution of the pro-inflammatory and pro-proliferative cytokine IL-6. To this end, IL-6 concentrations were measured in culture supernatants of L3.6pl cells after incubation with LPS and raloxifene. Raloxifene exposure for 24 hours resulted in a reduction of the LPS-induced IL-6 production. In comparison to the control, a decreasing amount of IL-6 was seen with increasing concentrations of raloxifene. The mean values of the IL-6 concentrations after 24 hours of raloxifene exposition are shown in Fig. 2a.

### Raloxifene suppresses IL-6/STAT3 signaling in a dose- and time-dependent manner

To test whether STAT3 phosphorylation levels could be stimulated by IL-6, L3.6pl cells were treated with IL-6. We found that IL-6 stimulated STAT3 phosphorylation in L3.6pl cells (Fig. 2b). The IL-6 induced constitutive phosphorylation of STAT3^Y705^ was effectively suppressed by raloxifene in a dose-dependent manner (Fig. 2b). Notably, raloxifene showed a maximal suppressive effect on STAT3^Y705^ phosphorylation after three hours of treatment and its effect was no longer detectable after 24 hours (Fig. 2c).

### Raloxifene binds specifically to gp130

A DARTS assay was performed to identify and assess a protein-ligand interaction between raloxifene and gp130 and the binding of SC144 to gp130. We found that SC144 and raloxifene bind specifically to gp130 in L3.6pl cell lysates, thereby reducing the protease susceptibility of gp130 (Fig. 2d).

### Raloxifene suppresses the growth of human PDAC xenografts

**Fig. 3:**
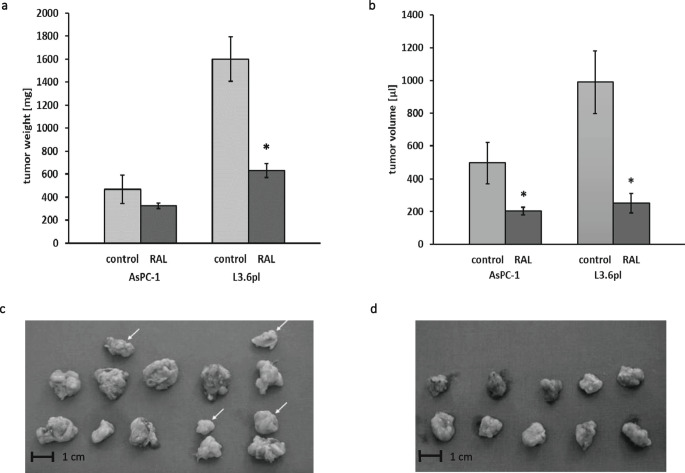
Raloxifene suppresses tumor growth in human pancreatic cancer xenografts. Raloxifene suppressed tumor weights (**a**) and tumor volumes (**b**) in L3.6pl and AsPC-1 nude mouse xenograft models (* *p* < 0.05). Macroscopy of primary pancreatic tumors (n = 10) and wound site tumors (arrows, n = 4) of the L3.6pl control group (**c**) and primary pancreatic tumors (n = 10) of the L3.6pl raloxifene group (**d**). Note the reduced tumor sizes and the absence of wound tumors in the raloxifene-treated mice

To determine the effect of raloxifene on PDAC progression in vivo, primary xenograft growth and the development of lymph node and liver metastases of AsPC-1 and L3.6pl cells were assessed. All animals developed primary orthotopic tumors. In AsPC-1 tumors, the mean tumor weight in raloxifene-treated mice was 325 mg (n = 6) and in controls 468 mg (n = 6, *p* = 0.151, Fig. 3a). The mean tumor volume was 202 µl in raloxifene-treated mice and 496 µl in controls (*p* = 0.044, Fig. 3b). In L3.6pl tumors, the mean tumor weight in raloxifene-treated mice was 631 mg and in controls 1600 mg (n = 10, *p* < 0.001, Fig. 3a). The mean tumor volume was 251 µl in the raloxifene-treated mice and 990 µl in controls (n = 10, *p* < 0.001, Fig. 3b). No significant body weight loss was detected during the treatment period.

### Raloxifene inhibits metastatic spread of PDAC in vivo

In case of AsPC-1 tumors, lymph node metastases were found in six mice, four in control mice (67%) and two in raloxifene-treated mice (33%). No liver metastases were detected. Splenic metastases were detected in one control mouse. Wound site tumors were also evaluated. Four of six control mice exhibited wound site tumors, whereas two of the mice in the treatment group exhibited wound site tumor growth. In case of L3.6pl tumors, liver metastases were detected in six mice, five in control mice (50%) and one in a raloxifene-treated mouse (10%). Extra-abdominal lymph node metastases were found in five mice. In two control mice, all recognizable lymph nodes were enlarged, whereas in another mouse swelling was limited to the axillary lymph nodes. In the raloxifene-treated group, enlarged axillary lymph nodes were found in one case, and inguinal lymph nodes were found to be affected in another case. Wound site tumors were also evaluated. Four of 10 control mice exhibited wound site tumors with weights ranging from 288 mg to 408 mg (arrows in Fig. 3c). None of the mice in the treatment group exhibited wound site tumor growth (Fig. 3d).

### Raloxifene suppresses PDAC cell proliferation in vivo and ERβ is expressed in pancreatic cancer xenografts

**Fig. 4:**
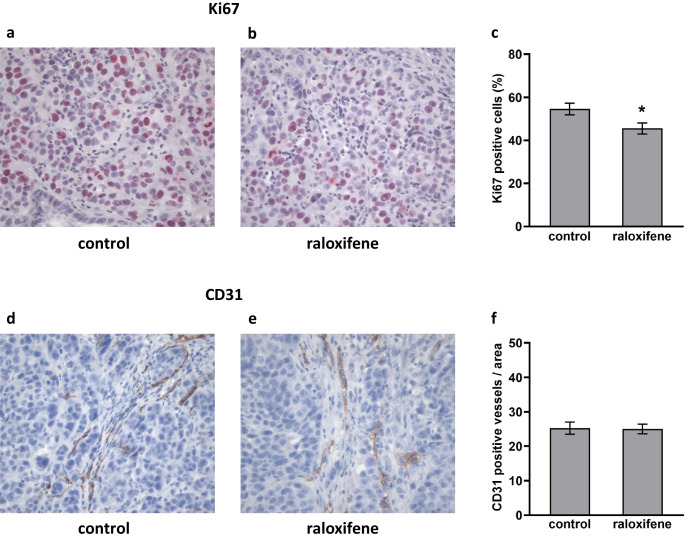
Raloxifene suppresses in vivo pancreatic cancer cell proliferation. IHC showing that the mean of Ki67 positive cells was 54.6% in the control group (**a** and **c**) and 45.5% in the treatment group (**b** and **c**); * *p* = 0.026. Raloxifene treatment did not affect tumor micro-vessel density, i.e., the mean of CD31-positive cells was similar in both groups. In the control group (**d** and **f**) the score was 25.3/area and in the raloxifene-treated group the score was 25.0/area (**e** and **f**; original magnification 400x)

**Fig. 5:**
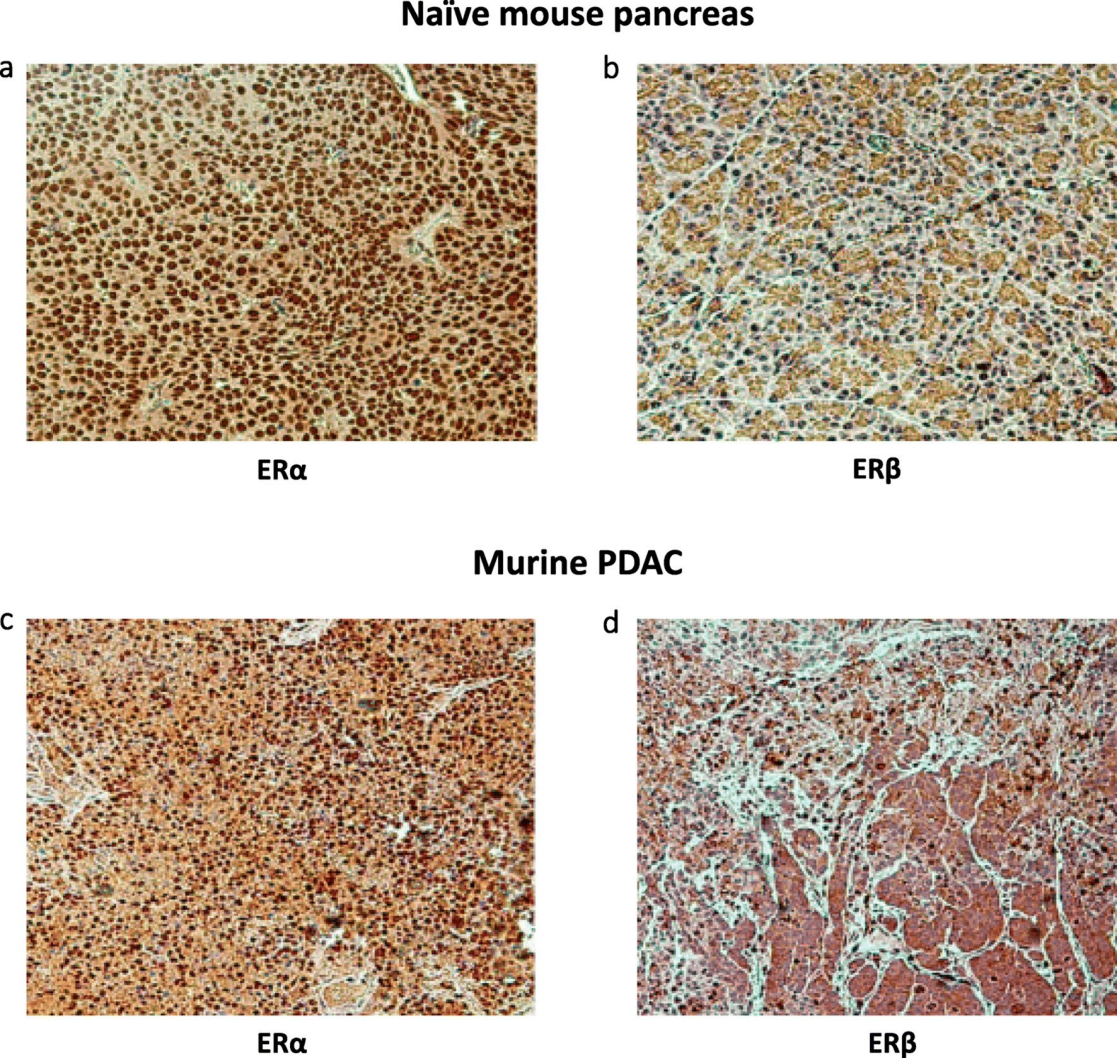
IHC of normal mouse pancreas and pancreatic cancer tissues. Normal mouse pancreatic tissues express ERα but not ERβ (**a** and **b**, respectively), whereas both ERα and ERβ are expressed in pancreatic cancer tissues (**c** and **d**, respectively); original magnification 200x

Expression of the proliferation marker Ki67 and the endothelial cell marker CD31 was analyzed in PDAC xenograft specimens (L3.6pl). In addition, the ERα and ERβ status of the tumors was evaluated. Consistent with our in vitro data, we found that raloxifene treatment suppressed PDAC cell proliferation in the orthotopic tumors (Fig. 4a, b and c). The mean of Ki67-positive cells was 45.5 ± 2.5% in raloxifene-treated mice and 54.6 ± 2.7% in controls (*p* = 0.026, Fig. 4c). Raloxifene treatment did not affect the tumor micro-vessel density, as revealed by CD31 staining (Fig. 4d and e). The score was 25.0 ± 1.4/area in raloxifene-treated mice, and 25.3 ± 1.8/area in controls (*p* = 0.914, Fig. 4f). Finally, we found that the normal pancreatic tissues of the mice express ERα but not ERβ (Fig. 5a and b, respectively). In contrast, both ERα and ERβ were found to be expressed in the pancreatic cancer tissues (Fig. 5c and d).

## Discussion

In the present study, we showed that the SERM raloxifene inhibits local and metastatic PDAC progression in an orthotopic xenograft model and suppresses PDAC cell proliferation in vitro and in vivo dependent on the presence of ERβ. In addition, we found that IL-6 expression in PDAC cells was diminished by raloxifene treatment as well as the phosphorylation of its downstream effector STAT3. A possible mechanism may be the binding of raloxifene to gp130.

ERs are expressed in various solid tumors. The relevance of ER expression in breast cancer is well established, and SERMs represent backbones in ER-positive breast cancer treatment [20]. Beside benign and malignant pancreatic neoplasms that have been shown to express ERα and ERβ, ER expression has also been observed in colorectal, gastric, esophageal and hepatocellular cancer [21–25]. The role of ER expression in these tumor entities, however, is less well defined compared to that in breast cancer, since ligand/ER interactions may be modified by affinity, transcriptional activation of genes, interaction with cofactors, heterogeneous receptor dimerization, ER splice variants and ERα/ERβ ratios [26]. By analyzing clinical tumor specimens of 175 PDAC patients, we previously found that ERβ and phosphorylated ERβ (pERβ) are highly expressed in the majority of pancreatic cancer patients (61.7% and 80.6%, respectively) and that pERβ expression correlates with a poor prognosis in these patients [12, 13]. This led to the question whether targeting ERβ would have an impact on PDAC tumor progression.

The benzothiophene raloxifene is FDA approved for clinical use and found to be effective in the treatment of osteoporosis and the prevention of breast cancer in high risk patients [27–29]. Raloxifene exerts its actions in a tissue-specific manner, binding with high affinity to both ERα and ERβ and acting as an ER agonist, antagonist, or both [30]. Here, we found that raloxifene exerts antiproliferative effects in vitro and in vivo and reduces the viability of PDAC cells. In line with our results, proliferation was previously found to be reduced by raloxifene in colon cancer cells in an ER-dependent manner [31, 32]. Similar effects were observed in breast cancer [33], hepatocellular carcinoma [34] and prostate cancer [35], as well as in non-cancer cell types [36]. In addition, we found that the inhibition of proliferation by raloxifene appeared to be at least in part dependent on the presence of ERβ but not ERα, since ERβ silencing, unlike ERα silencing, abolished the inhibitory effects induced by raloxifene, suggesting ERβ-mediated effects in PDAC cell proliferation. In line with these results, the presence of ERβ in non-small cell lung cancer has been found to increase proliferation that was inhibited by raloxifene, however this effect was seen only in the presence of estradiol, suggesting genomic effects via DNA sequences known as raloxifene responsive elements [37]. Since phosphorylated ERβ is present in 80% of PDAC patients, and acts as a negative prognostic factor, also non-genomic effects may affect ER-mediated PDAC progression [12]. The observation that ERα/ERβ ratios are lower in PDAC than in breast cancer patients [6] may suggest an important role of ERβ in PDAC.

More importantly, beside its partial ERα agonist/antagonist and ERβ antagonist actions, raloxifene may directly interfere with IL-6 signaling. More specifically, recent in silico evidence indicates that raloxifene can bind to gp130, which forms a complex with the IL-6 receptor to initiate downstream signaling involving the JAK/STAT3 pathway [38]. Our data show that raloxifene inhibits IL-6 synthesis in PDAC cells and, as a second mechanism of action, potently suppresses IL-6 induced STAT3 phosphorylation. IL-6 is crucial in KRAS driven PDAC development and progression from PanIn lesions to invasive cancer [39] and promotes cell viability and invasion, underlining its metastatic potential [40]. In patients with PDAC, elevated serum IL-6 levels have been found to be associated with poor survival [41]. In addition, tumor stroma-derived IL-6 has been shown to promote tumor angiogenesis and tumor-stroma interaction [42, 43]. STAT3 is known to be phosphorylated following IL-6 receptor activation and to serve as a negative prognostic factor in PDAC [44]. Similar to our results, STAT3 phosphorylation has been found to be suppressed by raloxifene in breast and colon cancer, thereby promoting apoptosis and decreasing cell viability [45]. Our results show that in PDAC cells raloxifene binds specifically to gp130 with an affinity similar to that of SC144, a specific inhibitor of gp130. In line with our results, gp130 inhibition with the SERM bazedoxifene has been reported to result in a reduction of interleukin-11 induced STAT3 phosphorylation, proliferation and viability, as well as organoid and xenograft growth in colon cancer [46, 47]. Similar results have been reported for hepatocellular cancer [48] and rhabdomyosarcoma [49]. Taken together, SERM interaction with the IL-6/STAT3 pathway through gp130 binding and inactivation independent of ER binding seems to be an important mechanism contributing to the antitumor effects of raloxifene in PDAC cells.

We found that raloxifene had no significant effect on endothelial cell count. Regarding tumor angiogenesis, current data for SERMs are conflicting. While there is evidence that tamoxifen inhibits platelet-mediated tumor angiogenesis [50], raloxifene has been found to increase the proliferation of endothelial cells in vitro [51]. Although our data cannot exclude anti-angiogenic properties of raloxifene in PDAC, their role in our experimental setting seems to be of limited relevance.

Raloxifene markedly reduced orthotopic and metastatic PDAC xenograft growth in both PDAC cell systems tested. This effect was more pronounced in the fast growing L3.6pl cell line. Although AsPC-1 xenograft tumor growth and metastatic spread were not as aggressive as those of L3.6pl tumors, the AsPC-1 tumor volumes in raloxifene treated mice were also significantly reduced in comparison to the control group. These findings are similar to recent data reporting a growth reduction by bazedoxifene in PDAC [52] and liver cancer [10] xenografts.

Clinical trials using tamoxifen as monotherapy or combined with gemcitabine in advanced pancreatic cancer have been controversial [10, 11]. An explanation may be that in these trials, the patients´ ER status was not taken into consideration. In clinical routine, assessment of ER expression in patients with breast cancer is a standard method to select the proper therapeutic approach with or without hormonal SERM therapy. The ER status may also be of importance for successfully treating PDAC patients with SERMs, as we found that the antiproliferative effects of raloxifene are at least partly dependent on the presence of ER. Moreover, no marked effect of tamoxifen on gp130/IL-6/STAT3 signaling, compared to raloxifene and bazedoxifene, has been reported [53]. In breast cancer models, bazedoxifene was found to act synergistically with the cytotoxic taxane paclitaxel. Translating this combination therapy (SERM and paclitaxel) into PDAC may be an attractive approach, since SERMs may antagonize increases in IL-6 in patients treated with paclitaxel, which may result in synergism.

In conclusion, we found that raloxifene potently inhibits PDAC progression in vitro and in in vivo orthotopic tumor cell xenografts through a mechanism that involves binding and inhibition of gp130 and, thereby, abrogation of IL-6 signaling. These findings may contribute to clinical application of SERMs alone or in combination with other compounds in PDAC.

## Abbreviations

ER: estrogen receptor
FDA: Food and Drug Administration
SERM: selective estrogen receptor modulator
ERβ: estrogen receptor beta
IL-6: interleukin-6
gp130: glycoprotein-130
BrdU: 5-bromo-2′-deoxyuridine
siRNA: small interfering ribonucleic acid
ERα: estrogen receptor alpha
ELISA: enzyme-linked immunosorbent assay
STAT3: signal transducer and activator of transcription 3
PDAC: pancreatic ductal adenocarcinoma
DMSO: dimethyl sulfoxide
PBS: phosphate buffered saline
BCA: bicinchoninic acid
DARTS: drug affinity responsive target stability
CD31: cluster of differentiation 31
PECAM-1: platelet endothelial cell adhesion molecule 1
pERβ: phosphorylated ERβ
hpf: high power field
RNAi: RNA interference
LPS: lipopolysaccharide
STAT3^Y705^: signal transducer and activator of transcripttion 3 phosphorylated at tyrosine 705
SD: standard deviation
