# Correction to: Raloxifene inhibits pancreatic adenocarcinoma growth by interfering with ERβ and IL-6/gp130/STAT3 signaling

**DOI:** 10.1007/s13402-025-01128-8

**Published:** 2025-12-08

**Authors:** Ioannis Pozios, Nina N. Seel, Nina A. Hering, Lisa Hartmann, Verena Liu, Peter Camaj, Mario H. Müller, Lucas D. Lee, Christiane J. Bruns, Martin E. Kreis, Hendrik Seeliger

**Affiliations:** 1 Department of General, Visceral and Vascular Surgery, Campus Benjamin Franklin, Charité Universitätsmedizin Berlin, Hindenburgdamm 30, 12203 Berlin, Germany; 2 Department of General, Visceral and Transplantation Surgery, Hospital of the University of Munich, Munich, Germany; 3 Department of Minimal Invasive and Visceral Surgery, Vivantes Klinikum Neukölln, Berlin, Germany; 4 Department of General, Visceral, Cancer and Transplant Surgery, University Hospital of Cologne, Cologne, Germany

**Correction to: Cellular Oncology (2020) 44:167–177**

10.1007/s13402-020-00559-9

Following the publication of this article it was noted that an incorrect immunostaining image for CD31-positive cells in the raloxifene-treated group was shown in panel 'e' of Figure 4. In the course of correcting this error, the entire Figure 4 has been revised, including updated immunostaining images and corresponding data for Ki67- and CD31-positive cells in both the control and raloxifene-treated groups.

Following Editor approval, Figure 4, as well as sections 2.8 and 3.8 of the article, have been updated accordingly. These corrections do not affect the overall conclusions of the article.

Incorrect originally published Figure 4:

**Fig. 4:**
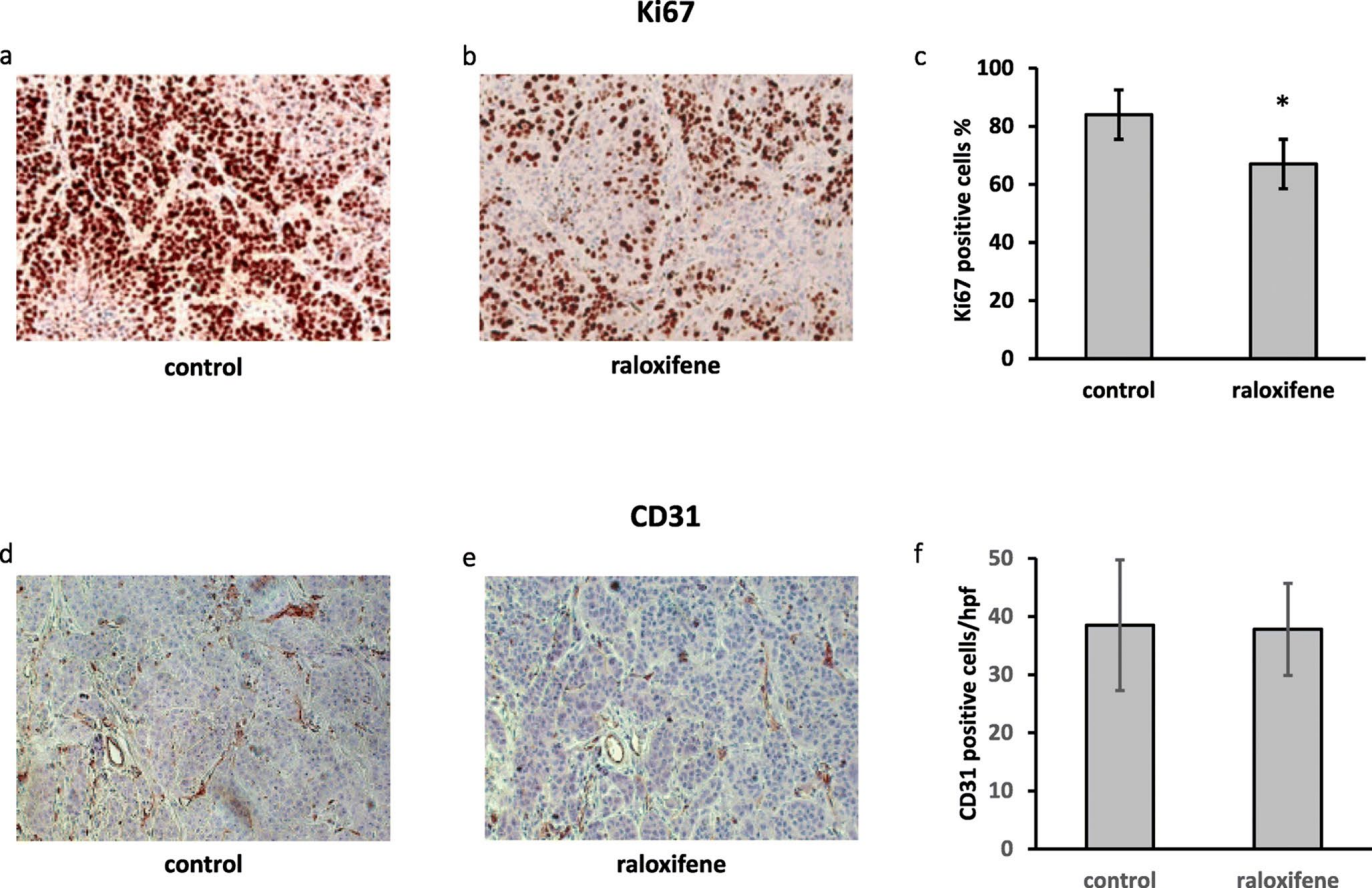
Raloxifene suppresses in vivo pancreatic cancer cell proliferation. IHC showing that the mean of Ki67 positive cells was 84% in the control group (**a**) and 67% in the treatment group (**b** and **c**); * *p* = 0.014. Raloxifene treatment did not affect tumor micro-vessel density, i.e., the mean of CD31-positive cells was similar in both groups. In the control group (**d**) the score was 38.5/hpf and in the raloxifene-treated group the score was 37.8/hpf (**e** and **f**; original magnification 200x)

Corrected Figure 4:

**Fig. 4:**
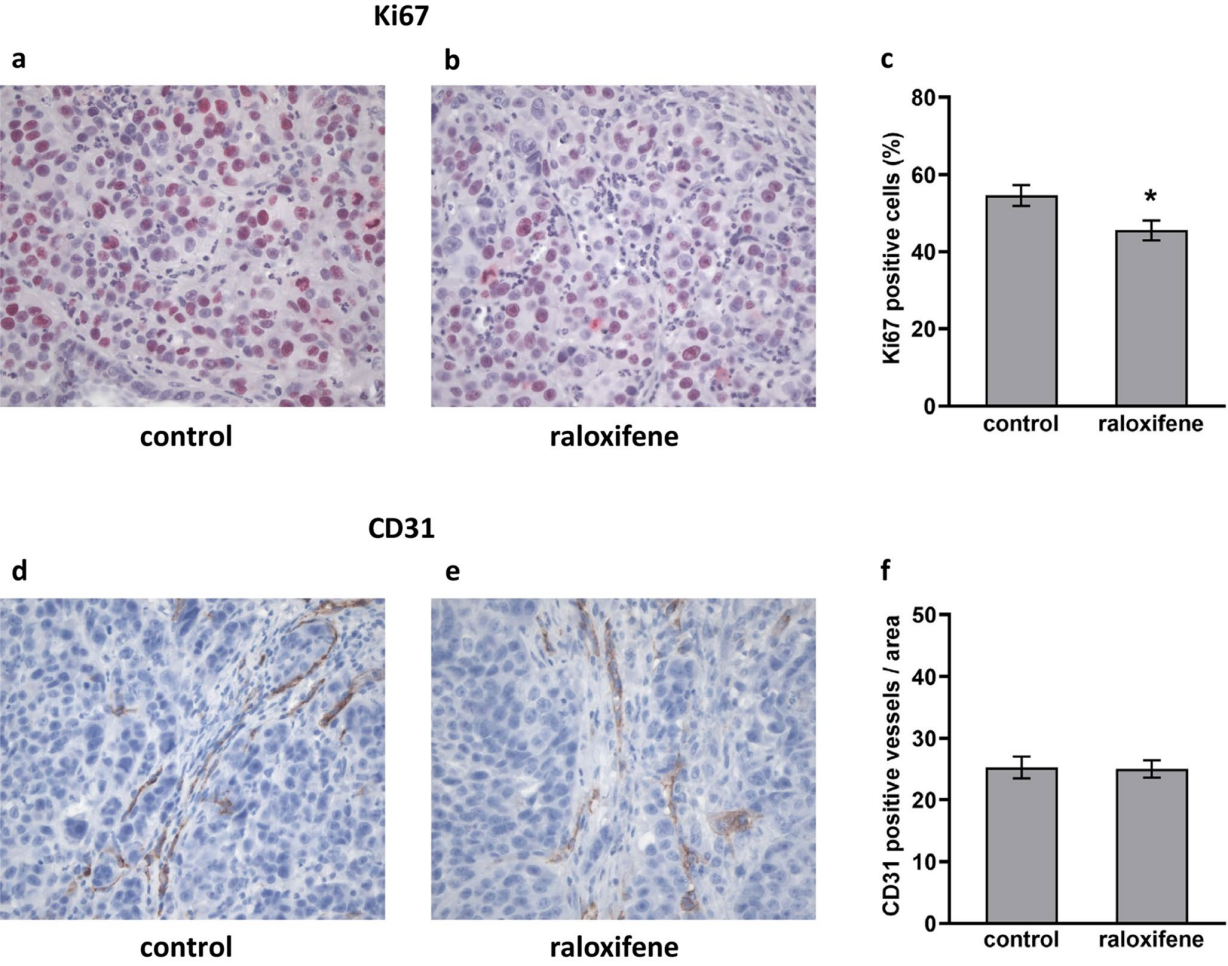
Raloxifene suppresses in vivo pancreatic cancer cell proliferation. IHC showing that the mean of Ki67 positive cells was 54.6% in the control group (**a** and **c**) and 45.5% in the treatment group (**b** and **c**); * *p* = 0.026. Raloxifene treatment did not affect tumor micro-vessel density, i.e., the mean of CD31-positive cells was similar in both groups. In the control group (**d** and **f**) the score was 25.3/area and in the raloxifene-treated group the score was 25.0/area (**e** and **f**; original magnification 400x)

*Incorrect originally published text of section 2.8 Immunohistochemistry (IHC):*

Paraffin-embedded tissues were used for the assessment of Ki67, CD31 (PECAM-1), ERα and ERβ expression. Anti-ERα, anti-ERβ, anti-Ki67 and anti-CD31 antibodies were purchased from Abcam (Cambridge, UK). All sections were first deparaffinized in Neo-Clear™ (Merck, Darmstadt, Germany) and rehydrated in a graded series of alcohol. Tissue sections were subsequently blocked by 8% bovine serum albumin using an Avidin/Biotin blocking kit (Vector Laboratories, Burlingame, CA, USA), followed by incubation with the indicated antibody at 4 °C overnight. Next, tissue sections were incubated with a biotin-conjugated secondary antibody (Dako GmbH, Hamburg, Germany) using an Avidin-Biotin complex kit (Vector Laboratories, Burlingame, CA, USA). DAB + chromogen (3,3 diaminobenzidine; Dako GmbH, Hamburg, Germany) was used to generate fluorescent signals, whereas counterstaining was carried out using a hemalum solution (Merck, Darmstadt, Germany). Subsequent immunoperoxidase-based detection was performed using a LSAB2 System (Dako GmbH, Hamburg, Germany), followed by staining using an AEC kit system (Zytomed Systems GmbH, Berlin, Germany).

For quantification of Ki67 staining, the number of Ki67 positive cells was scored using a range from zero to four according to the percentage of positive cells. Percentages were determined by the number of stained cells divided by the number of nuclei. For the quantification of microvessel density, pancreatic tumor sections were fixed and stained with antibodies directed against CD31. For quantification of CD31 expression, the number of endothelial cells was counted in two fields of 0.159-mm^2^ each at 100x magnification in the areas exhibiting most intense CD31 expression, and the mean was calculated as the number of microvessels pro high power field (hpf = 0,159 m^2^).

*Corrected text of section 2.8 Immunohistochemistry (IHC):*

Paraffin-embedded tissues were used for the assessment of Ki67, CD31 (PECAM-1), ERα and ERβ expression. Anti-ERα, anti-ERβ and anti-Ki67 antibodies were purchased from Abcam (Cambridge, UK), anti-CD31 was purchased from Cell Signalling (Cambridge, UK). 

All sections were first deparaffinized in Neo-Clear™ (Merck, Darmstadt, Germany) and rehydrated in a graded series of alcohol. Tissue sections were subsequently blocked by 8% bovine serum albumin using an Avidin/Biotin blocking kit (Vector Laboratories, Burlingame, CA, USA), followed by incubation with the indicated antibody at 4 °C overnight. Next, tissue sections were incubated with a biotin-conjugated secondary antibody (Dako GmbH, Hamburg, Germany) using an Avidin-Biotin complex kit (Vector Laboratories, Burlingame, CA, USA). DAB + chromogen (3,3 diaminobenzidine; Dako GmbH, Hamburg, Germany) was used to generate fluorescent signals, whereas counterstaining was carried out using a hemalum solution (Merck, Darmstadt, Germany). Immunoperoxidase-based detection was performed using HistoMark^®^ RED (Sera Care, Milford, USA). 

Microscopy was performed using a 400x magnification and ten fields of each section were pictured by Axiocam 305 color (Zeiss, Jena, Germany). The area of each pictured field was 0.0952 mm^2^. For quantification of Ki67 staining, the number of Ki67 positive and negative cells were counted in each field and percentages of positive cells were calculated. For the quantification of microvessel density, the number of vessels was counted in ten areas exhibiting most intense CD31 expression. The mean was calculated as the number of microvessels per area (0.0952 mm^2^). All values are given as mean ± SEM.

*Incorrect originally published text of section 3.8 Raloxifene suppresses PDAC cell proliferation in vivo and ERβ is expressed in pancreatic cancer xenografts:*

Expression of the proliferation marker Ki67 and the endothelial cell marker CD31 was analyzed in PDAC xenograft specimens (L3.6pl). In addition, the ERα and ERβ status of the tumors was evaluated. Consistent with our in vitro data, we found that raloxifene treatment suppressed PDAC cell proliferation in the orthotopic tumors (Fig.  4a, b and c). The mean index of Ki67-positive cells was 3.0 (67% positive cells) in raloxifene-treated mice and 3.6 (84%) in controls (*p* = 0.014, Fig. 4c). Raloxifene treatment did not affect the tumor micro-vessel density, as revealed by CD31 staining (Fig. 4d and e). The score was 37.8/hpf in raloxifene-treated mice, and 38.5/hpf in controls (Fig. 4f). Finally, we found that the normal pancreatic tissues of the mice express ERα but not ERβ (Fig. 5a and b, respectively). In contrast, both ERα and ERβ were found to be expressed in the pancreatic cancer tissues (Fig. 5c and d).

*Corrected text of section 3.8 Raloxifene suppresses PDAC cell proliferation in vivo and ERβ is expressed in pancreatic cancer xenografts:*

Expression of the proliferation marker Ki67 and the endothelial cell marker CD31 was analyzed in PDAC xenograft specimens (L3.6pl). In addition, the ERα and ERβ status of the tumors was evaluated. Consistent with our in vitro data, we found that raloxifene treatment suppressed PDAC cell proliferation in the orthotopic tumors (Fig. 4a, b and c). The mean of Ki67-positive cells was 45.5 ± 2.5% in raloxifene-treated mice and 54.6 ± 2.7% in controls (*p* = 0.026, Fig. 4c). Raloxifene treatment did not affect the tumor micro-vessel density, as revealed by CD31 staining (Fig. 4d and e). The score was 25.0 ± 1.4/area in raloxifene-treated mice, and 25.3 ± 1.8/area in controls (*p* = 0.914, Fig. 4f). Finally, we found that the normal pancreatic tissues of the mice express ERα but not ERβ (Fig. 5a and b, respectively). In contrast, both ERα and ERβ were found to be expressed in the pancreatic cancer tissues (Fig. 5c and d).

